# Atomic Structure and Electronic Properties of Janus SeMoS Monolayers on Au(111)

**DOI:** 10.1021/acs.nanolett.4c06543

**Published:** 2025-02-18

**Authors:** Julian Picker, Mahdi Ghorbani-Asl, Maximilian Schaal, Silvan Kretschmer, Felix Otto, Marco Gruenewald, Christof Neumann, Torsten Fritz, Arkady V. Krasheninnikov, Andrey Turchanin

**Affiliations:** † Institute of Physical Chemistry, Friedrich Schiller University Jena, 07743 Jena, Germany; ‡ Institute of Ion Beam Physics and Materials Research, Helmholtz-Zentrum Dresden-Rossendorf, 01328 Dresden, Germany; § Institute of Solid State Physics, Friedrich Schiller University Jena, 07743 Jena, Germany; ∥ Abbe Center of Photonics, Friedrich Schiller University Jena, 07745 Jena, Germany

**Keywords:** Janus monolayers, transition metal dichalcogenides, 2D materials, electronic structure, chemical vapor deposition

## Abstract

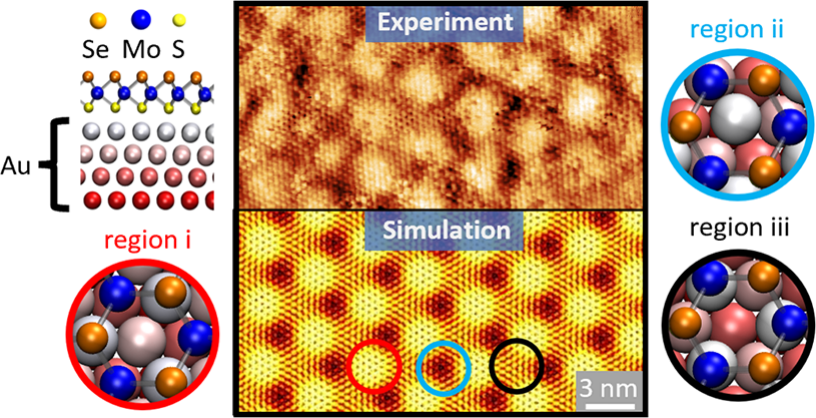

Janus SeMoS monolayers (MLs) are synthetic 2D materials with unique electronic properties, as theory predicts, but their experimental exploration has been hindered by the low quality of the samples. Here we report a synthesis of high-quality Janus MLs on gold substrates by thermal exchange reaction taking place at the ML/Au(111) interface. The synthesized Janus SeMoS MLs were characterized by complementary techniques, and insights into the topography and electronic properties of the system were obtained. Specifically, due to the lattice mismatch with the Au(111), a moiré pattern with a periodicity of 2.9 nm was observed. A precise experimental determination of the lattice constant of Janus SeMoS of 3.22 ± 0.01 Å was obtained, and the measured spin–orbit splitting at the *K* point of the valence band was found to be 170 ± 15 meV, matching well the results of the density functional theory calculations.

Transition metal dichalcogenide (TMD) monolayers (MLs) including 1H MoS_2_, MoSe_2_, WS_2_, WSe_2_, *etc.* are two-dimensional (2D) materials with unique electronic and photonic properties continuously attracting a significant research interest.^1,2^ Recently, a new class of TMD MLs, the so-called Janus TMDs, came into the focus of research.^3−6^ In contrast to the conventional TMD MLs, in which transition metal atoms are sandwiched between chemically similar chalcogens, in Janus TMD MLs the chalcogen atoms differ between the different faces of a ML. This asymmetry results in the broken out-of-plane mirror symmetry and leads to an intrinsic dipole which may cause new physical properties.^7^ Numerous theoretical studies of Janus TMDs predict strong Rashba splitting,^8,9^ piezoelectric,^10,11^ catalytic,^12^ novel excitonic,^13^ valleytronic^14^ and spintronic phenomena,^15^ which have to be proven experimentally yet. Experimental studies of Janus TMD MLs are still limited (see, e.g., refs (16−26)). Unlike conventional TMD MLs, which can be exfoliated from bulk crystals, these nanomaterials can only be obtained by bottom-up synthetic approaches, which limit their availability to the experimentalists. The reported synthesis methods are often based on complex methodologies including stripping of the topmost chalcogen layer of TMD ML by laser pulses,^18,26^ H_2_ plasma^19^ and refilling of the formed vacancies with chalcogen atoms of the second type. Such approaches typically result in a high defect density in the formed Janus TMD MLs, which complicates the revealing of the theoretically predicted properties. Beyond Janus TMD monolayers several other Janus materials with distinct electronic properties have been studied so far including Janus IV–VI structures,^27,28^ Janus transition metal trichalcogenide monolayers,^29^ Janus transition metal dichalcogenide oxides,^30^ or Janus transition metal oxyhalides.^31^

Recently, a method for the large-area synthesis of high-quality Janus SeMoS MLs was introduced, which is based on the exchange of the chalcogen atoms of the parent TMD ML at the ML/substrate interface via intercalation of chalcogen atoms of the second type.^16^ In this method, a MoSe_2_ ML is first grown on gold foils by chemical vapor deposition (CVD). Next, this monolayer is exposed to sulfur vapor at elevated temperatures. Due to the high affinity of sulfur to gold, sulfur atoms can intercalate between the MoSe_2_ ML/gold interface leading to an exchange of the bottom Se layer by S and the formation of a SeMoS ML, as schematically depicted in Figure 1a. The quality of the formed Janus SeMoS MLs is characterized at low temperatures by a narrow photoluminescence line width of only 18 meV, high circular polarization of the excitonic emission as well as by the valley Zeeman splitting.^16^

**Figure 1:**
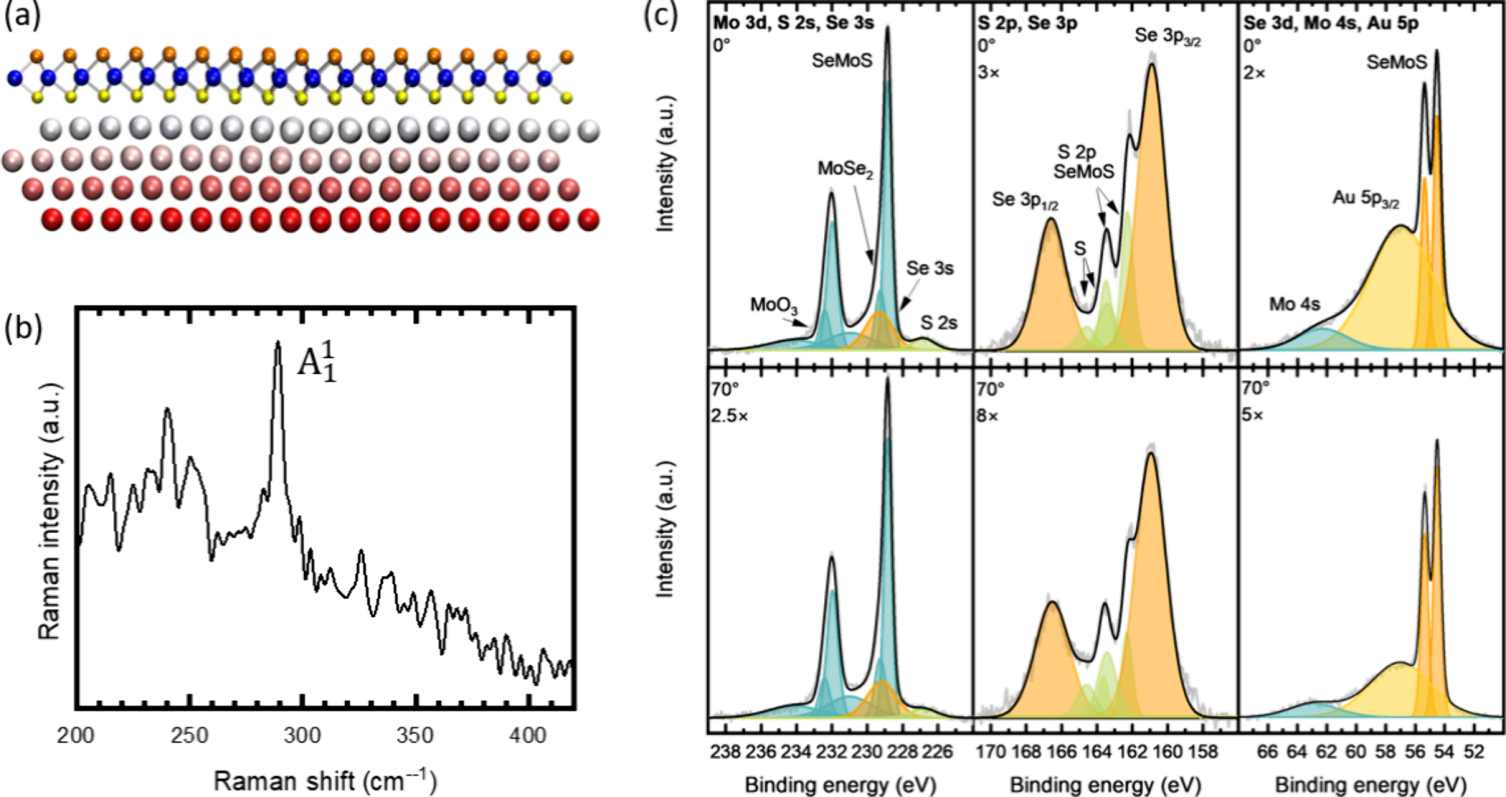
(a) Cross section of Janus SeMoS ML on Au(111) based on DFT calculations. Selenium, molybdenum, and sulfur atoms are highlighted in orange, blue, and yellow, respectively. The gold substrate atoms are represented as larger balls and colored according to their depth. (b) Raman spectrum (excitation wavelength, 532 nm) and (c) Mo 3d/S 2s/Se 3s (left), S 2p/Se 3p (middle), and Se 3d/Mo 4s/Au 5p (right) XP spectral regions of Janus SeMoS on Au(111) recorded at an emission angle of 0° (top) and 70° (bottom). Mo, S, Se, and Au peaks are highlighted in blue, green, orange, and yellow, respectively.

Here we present the structural and electronic characterization down to the nanoscale of the grown Janus SeMoS MLs on Au(111). Due to the lattice mismatch between the Janus SeMoS and Au(111), we observe a moiré superstructure by scanning tunneling microscopy (STM) and low-energy electron diffraction (LEED) and precisely determine the lattice parameters of the Janus SeMoS from the LEED pattern. Further, we measure the band structure by angle-resolved ultraviolet photoelectron spectroscopy (ARUPS) and extract the value of the spin–orbit splitting of the valence band at the *K* point. To the best of our knowledge, such structural and electronic properties of the Janus TMD MLs are obtained experimentally for the first time. Our findings are backed by density functional theory (DFT) calculations on the SeMoS/Au(111) system.

The synthesis of Janus SeMoS MLs on Au(111) starts with the growth of MoSe_2_ MLs on Au(111) by ambient pressure CVD. Afterward, the as-grown MoSe_2_ MLs were sulfurized at 700 °C to exchange the Se layer at the Au(111) interface with S atoms. Details regarding the preparation of Janus SeMoS are given in the Experimental Section. To prove the formation of Janus SeMoS on Au(111) we carried out Raman spectroscopy measurements at ambient conditions. The spectrum shown in Figure 1b reveals a characteristic peak at ∼290 cm^–1^ corresponding to the *A*_1_^1^ mode of the Janus SeMoS.^16,32^

Next, we introduced the sample into a UHV chamber and studied it by angle-resolved X-ray photoelectron spectroscopy (XPS) measurements. The relevant XP spectra measured with an emission angle of 0° (top) and 70° (bottom) are shown in Figure 1c. A detailed peak analysis is presented in Table S1. First, we focus on the XP spectra measured at normal emission. In the Mo 3d region we observe a sharp, high intensity doublet at binding energies (BEs) of 228.9 ± 0.1 eV (Mo 3d_5/2_) and 232.0 ± 0.1 eV (Mo 3d_3/2_), which is due to the formed Janus SeMoS ML (see Figure 1c, left). The other low intensity Mo doublets, at BEs of 229.3 ± 0.1 eV/232.5 ± 0.1 eV and 231.0 ± 0.2 eV/234.2 ± 0.2 eV, originate from remaining nonconverted MoSe_2_ and MoO_3_, respectively. Since the intensity of the Janus SeMoS features is about 4 times higher than that of the MoSe_2_ features, we conclude that about 80% of the as-grown MoSe_2_ is converted into Janus SeMoS. In the BE region of 171–156 eV (see Figure 1c, middle), dominated by the Se 3p peaks, a high intensity S 2p doublet at the BEs of 162.2 ± 0.1 eV (S 2p_3/2_) and 163.4 ± 0.1 eV (S 2p_1/2_), corresponding to the formed Janus SeMoS ML, is clearly recognized, which is accompanied by an additional low intensity S 2p doublet at a higher BE resulting most probably from excess unreacted sulfur clusters.^16,33^ In comparison to the S 2p peaks, the Se 3p peaks have a larger spin–orbit splitting and a broader full width at half-maximum. The Se species result also in the 3d doublet at BEs of 54.5 ± 0.1 eV (Se 3d_5/2_) and 55.4 ± 0.1 eV (Se 3d_3/2_) (see Figure 1c, right). In comparison to the Mo 3d spectrum, one cannot discriminate Se in the Janus SeMoS and MoSe_2_ MLs in this spectrum. However, if we consider the SeMoS to MoSe_2_ ratio obtained from the Mo 3d spectrum, we can estimate the amount of Se related to the SeMoS ML to be ∼70%. This estimation results in an elemental ratio for Se/Mo/S of (1.4 ± 0.3):1.0:(1.2 ± 0.2), which is in good agreement with the expected one of 1:1:1. The presence of the Se atoms on top and of the S atoms on the bottom of the SeMoS ML is further confirmed by the angular dependence XP intensities. Due to the surface sensitivity of XPS, as seen in Figure 1c and Table S1, the intensity ratio of Se/S is higher for an emission angle of 70° compared to normal emission (0°). These results conclusively confirm the formation of the SeMoS ML with its S face oriented toward the Au(111) substrate.

Next, we characterize the structural properties of the Janus SeMoS ML on Au(111) by LEED measurements. The distortion-corrected LEED pattern presented in Figure 2a shows a 6-fold symmetry. The reciprocal unit cells and the LEED spots of the Janus SeMoS and Au(111) are marked in cyan and red, respectively. The first-order spots of the Janus SeMoS reveal the highest intensity. Around each of these spots, six further spots are visible. As we demonstrate below, such a LEED pattern arises from a moiré structure, due to the lattice mismatch between the Janus SeMoS ML and the Au(111) substrate. Note that the formation of moiré structures is also observed for MoS_2_ and MoSe_2_ on Au(111).^34^ From the distortion-corrected LEED pattern, we precisely determined the lattice parameters of Janus SeMoS MLs on Au(111). For this quantitative analysis, we fitted all visible spots considering multiple scattering with the hexagonal structure of Au(111) with a lattice constant of 2.884 Å^35^ as a reference. The obtained lattice constant, a_SeMoS_, and the enclosing angle between both lattice vectors ∠(a⃗_1_,a⃗_2_) are 3.22 ± 0.01 Å and 120.00 ± 0.02°, respectively. Interestingly, the value of a_SeMoS_ is between the experimental values for MoS_2_ (3.15 ± 0.01 Å) and MoSe_2_ (3.28 ± 0.01 Å) MLs on Au(111) which were determined in the same way previously.^36^ The optimized freestanding SeMoS ML structure (without Au(111) substrate) calculated by DFT results in the lattice parameters of |a⃗_1_| = |a⃗_2_| = 3.22 Å = a_SeMoS_ and ∠(a⃗_1_,a⃗_2_) = 120° which are in a perfect agreement with our experimental results and previous theoretical reports.^37^ We further compared the values of the Janus SeMoS ML with the DFT-calculated values of freestanding MoS_2_ and MoSe_2_ MLs (see Table S2). Therewith, the optimized lattice constant of SeMoS (3.22 Å) lies between the values of MoS_2_ (3.15 Å) and MoSe_2_ (3.28 Å) and matches the experimental values obtained from LEED analysis very well. In the Janus ML, the Mo–S bond distances become longer by 0.01 Å than the corresponding bonds in a MoS_2_ ML, whereas the Mo–Se bond gets shorter by 0.01 Å than the corresponding bonds in MoSe_2_ monolayers. At the same time, the angle of Se–Mo–S bonds falls between those values obtained for the pristine materials. We calculated atomic Bader charges and averaged the electrostatic profile perpendicular to the Janus monolayer and compared it with that in the MoS_2_ and MoSe_2_ monolayer (Figures S1, S2 and Table S3). The Mo atoms became more positive in the SeMoS ML compared with MoS_2_, while the S atoms became more negative. On the other hand, the Se atoms have less negative charge than those in a MoSe_2_ ML. This leads to a higher charge density around the S atoms than the Se atoms as shown in Figure S1b. The electrostatic potential variation at different chalcogen layers is consistent with the charge transfer from the Mo to S or Se atoms. These results suggest a higher polarization in the Janus material in comparison with the pristine MoS_2_ and MoSe_2_ MLs.

**Figure 2:**
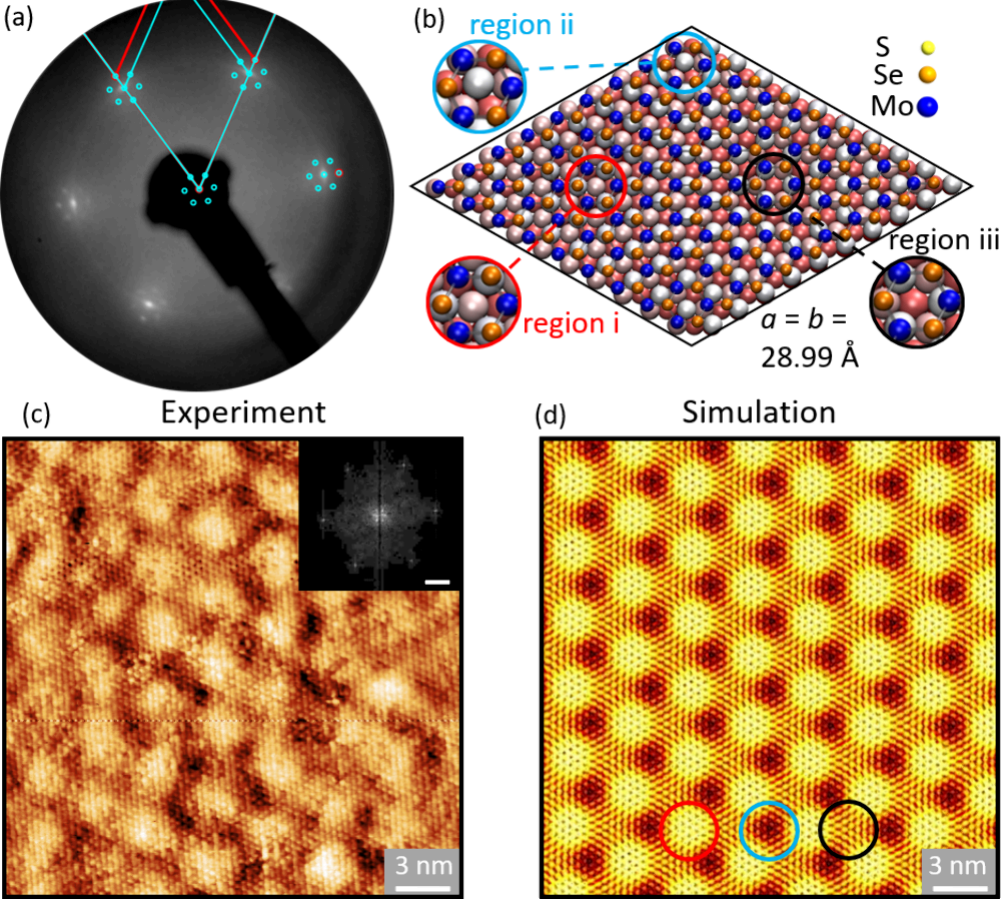
(a) LEED pattern of a Janus SeMoS ML on Au(111) (118 eV, 293 K). The unit cell and the corresponding LEED spots of the Janus SeMoS and Au(111) are highlighted in light blue and red, respectively. (b) Top view of an atomic ball model of the Janus SeMoS ML on Au(111). Due to the different adsorption sites of the sulfur layer with respect to the topmost Au layer, a moiré contrast appears. We distinguish three different regions which are discussed in the text. (c) STM (−1.1 V, 0.5 nA, 4.2 K) and (d) simulated STM images of a Janus SeMoS ML on Au(111). A fast Fourier transform (FFT) of the STM image in panel c is shown in the inset (scale bar ≈ 1 Å^–1^).

In Figure 3, we compare the structural models of MoS_2_, Janus SeMoS, and MoSe_2_ on Au(111). We simulated the lattices of MoS_2_ (blue), Janus SeMoS (cyan) and MoSe_2_ (green) and presented them with respect to the lattice of Au(111) (red). For the simulation, we used the following lattice constants obtained from LEED analysis: 3.15 Å^36^ (MoS_2_), 3.22 Å (Janus SeMoS), and 3.28 Å^36^ (MoSe_2_). These values lie within the uncertainties of the LEED analyses. The lattice constant of Au(111) is 2.884 Å.^35^ Due to the lattice mismatches between TMDs and Au(111) we observe different moiré structures. Although the lattice constants of the TMDs differ only slightly from each other, we observe more distinct differences in the corresponding moiré patterns, which allows for a more precise determination of the lattice constants. The moiré lattice constants are 34.6 Å for MoS_2_/Au(111), 28.9 Å for Janus SeMoS/Au(111), and 23.1 Å for MoSe_2_/Au(111). Thereby, an (11 × 11) MoS_2_ supercell matches very well a (12 × 12) Au(111) supercell.^38^ A (9 × 9) Janus SeMoS supercell matches very well a (10 × 10) Au(111) supercell. The same is true for a (7 × 7) MoSe_2_ and an (8 × 8) Au(111) supercell. In addition, we optimized the structure of the Janus SeMoS ML on Au(111) using DFT and extracted a moiré periodicity of 28.99 Å. This result is in excellent agreement with our estimated moiré periodicity based on LEED data.

**Figure 3:**
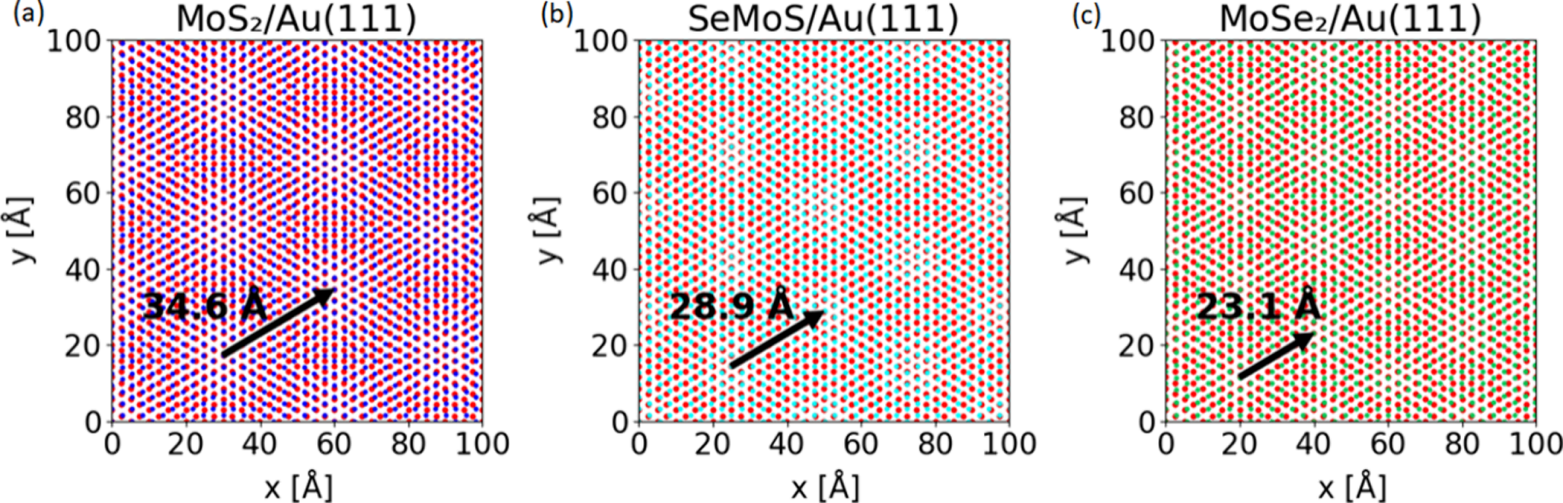
Representation of the lattices of (a) MoS_2_ (3.15 Å,^36^ blue), (b) Janus SeMoS (3.22 Å, light blue), and (c) MoSe_2_ (3.28 Å,^36^ green) on Au(111) (2.884 Å,^35^ red). Due to the lattice mismatch between the TMD and Au(111) a moiré contrast appears. The small differences in the lattice constant of the different TMDs results in large differences in the moiré lattice constant periodicities which are 34.6 Å for MoS_2_/Au(111), 28.9 Å for Janus SeMoS/Au(111), and 23.1 Å for MoSe_2_/Au(111).

To probe the Janus SeMoS structure on Au(111) in real space, we carried out low-temperature STM measurements. An atomically resolved STM image with the moiré contrast is shown in Figure 2c. The fast Fourier transform of the STM image confirms the hexagonal structure (FFT, inset of Figure 2b). We estimated the moiré periodicity from STM line scans to be ∼2.9 nm in agreement with the DFT calculations. Note that we analyzed the original STM data without any drift correction. Furthermore, an STM image was simulated by integrating occupied states within a narrow energy window (−0.1 eV) below the valence band edge which is shown in Figure 2d. The energy range of the simulated STM images can only be roughly compared to the bias voltages in the experiment due to the differences in the simulated and experimental bandgaps and the different zero energy levels, which corresponds to the Fermi level in the experiments and the highest occupied energy level in the calculations. Nevertheless, the simulated STM image displays good agreement with the experimental one. In the moiré unit cell, we can distinguish three different regions with slightly different electronic properties. These regions differ in the adsorption sites of the bottom S layer to the Au(111) atoms: (i) S atoms on top sites of Au(111), (ii) S atoms on fcc hollow sites, (iii) S atoms on hcp hollow sites. Region (i) has the largest STM height and appears brightest in the STM image. Regions (ii) and (iii) exhibit a significantly darker contrast whereby the STM height of region (iii) is slightly higher than for region (ii). The topography of the optimized Janus ML was carefully analyzed. The structure of SeMoS exhibits a slight buckling on Au(111) in different moiré regions of the interface. As a result, the equilibrium distance between SeMoS and substrate in region (i), i.e., 2.51 Å, is smaller compared to region (ii) (2.61 Å) and region (iii) (2.66 Å), suggesting stronger interaction between the lower S layer and Au atoms in region (i) than other regions. These results are consistent with the experimental STM image indicating high tunneling contrast in region (i).

Furthermore, we also observed point defects as shown in the STM image in Figure S3. These are likely Se vacancies in the top layer. We carefully evaluated the defect density, which proved to be about 10^12^ vacancies/cm^2^ confirming the high quality of our Janus SeMoS samples. To help identify the defects, STM images are simulated for Se vacancies in a SeMoS ML (Figure S4). The electronic structure characterization shows that Se vacancies lead to the formation of several in-gap states, including those being close to the conduction and valence band edges of the pristine SeMoS ML. The simulated image shows a single depression at the Se-vacancy site, which agrees well with the experimental STM data.

The electronic structure of Janus SeMoS MLs was characterized using DFT and ARUPS. The calculated band structure of a freestanding Janus SeMoS (without the Au(111) substrate) is shown in Figure 4a. We observe the valence band maximum (VBM) and the conduction band minimum (CBM) at the *K* point. This finding identifies the Janus SeMoS ML as a direct band semiconductor with a calculated band gap of 1.57 eV, which aligns closely with the previously reported theoretical value of 1.56 eV.^37^ The spin–orbit coupling (SOC) leads to a split of the valence and conduction band at the *K* point (Figure 4a). The energetic distance of the split valence bands is about 168 meV. Our calculated SOC splitting for MoS_2_ and MoSe_2_ was found to be 147 and 184 meV, respectively. The projected densities of states suggest that the VBM and CBM mainly result from Mo 4d and chalcogen p orbitals, whereas the sulfur orbitals have a larger contribution than the selenium states at the VBM (Figure S5). Specifically, the valence band splitting at the *K* point is primarily attributed to the in-plane Mo 4d_*xy*_ and Mo 4d_*x*^2^–*y*^2^_ orbitals.^39,40^ We also analyzed the effect of the substrate on the electronic structure of the Janus monolayer. The calculated interlayer binding energy of SeMoS on Au (111) is 1.1 J/m^2^, similar to those for MoS_2_ on metal substrates.^41^ A comparison between the projected DOS of freestanding and Au-supported SeMoS indicates that the interaction with the Au surface perturbs the electronic states of SeMoS close to the Fermi level as shown in Figure S4. It is also evident that the presence of the substrate shifts the Fermi level toward the conduction band minimum, suggesting a charge transfer at the interface. The calculated charge transfer from the substrate to SeMoS is presented in the charge density difference plots and is analyzed for each moiré region using Bader analyses (Figure 5a). The variation of the charge transfer at different moiré regions is caused by the different interactions between S atoms and Au atoms underneath (Figure 5b). Overall, the results indicate an electron transfer from gold to the Janus ML which is consistent with the observed n-type behavior of SeMoS in our electronic structure calculation (Figure S6).

**Figure 4:**
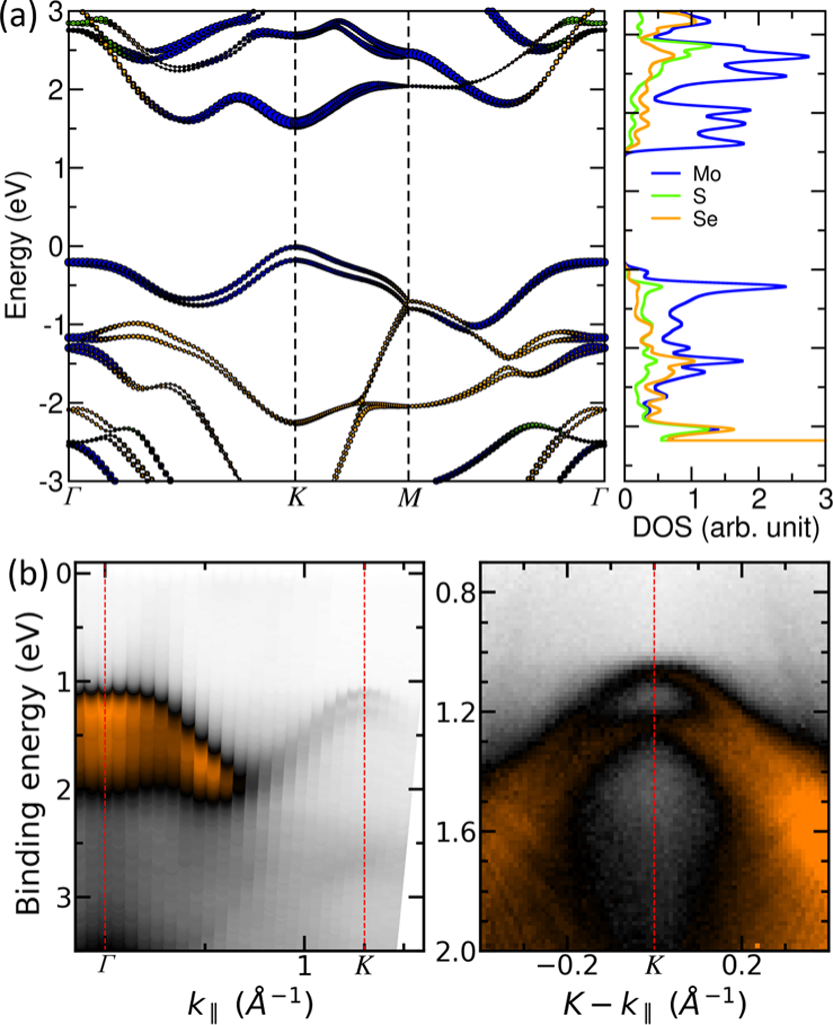
(a) Electronic band structure and the corresponding density of states of Janus SeMoS MLs calculated by DFT. (b) ARUPS data along the *Γ–K* direction and around the *K* point of Janus SeMoS MLs on Au(111).

**Figure 5:**
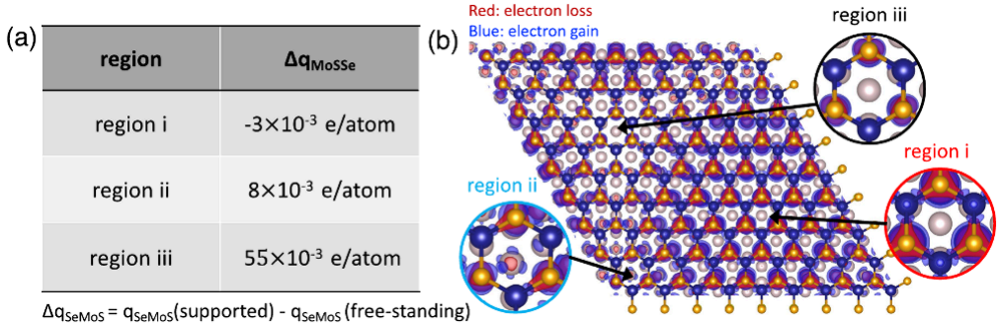
(a) Average Bader charges for all atoms at each moiré region in the SeMoS/Au(111) interface, as compared to the free-standing SeMoS monolayer. (b) Charge transfer between SeMoS and Au(111) calculated as the difference between the charges in the interface and the isolated Au(111) and SeMoS monolayer. Blue (red) colors indicate charge accumulation (depletion).

In Figures 4b and S7, the band structure of Janus SeMoS MLs on Au(111) measured by ARUPS is shown. From energy distribution curves (EDCs) at *Γ* and *K* points, we estimate the valence band maxima to be at BEs of 1.20 ± 0.10 eV and 1.06 ± 0.10 eV, respectively. These results mean that the valence band maximum has a slightly lower BE at the *K* point than at the *Γ* point which agrees with the DFT calculations. By zooming in the band structure (right part of Figure 4b), the spin–orbit split valence band at the *K* point can be recognized even more clearly. The splitting is 170 ± 15 meV in agreement with the DFT calculations of freestanding Janus SeMoS. From the maxima plot of each EDC as a function of their *k* value (Figure S8), we estimate the effective mass m* of the electron in the valence band to be 0.99 ± 0.10 m_e_. Using the calculated electronic band structure, the effective masses of the electron and the hole are calculated to be 0.60 m_e_ and 0.54 m_e_, respectively. Due to substrate interaction and hybridization, the measured effective mass of electrons in the valence band of Janus SeMoS MLs on Au(111) is larger than the calculated effective mass for freestanding Janus SeMoS.

The electronic structure of Janus SeMoS monolayers was characterized for the first time using ARUPS, enabling experimental determination of spin–orbit splitting and valence band effective mass, which are critical for understanding its electronic properties. DFT calculations further reveal that different regions of the Janus SeMoS monolayer interact distinctly with the Au(111) substrate, highlighting the complexity of substrate effects on its electronic structure.

In summary, we grew Janus SeMoS MLs with a high structural quality on Au(111) by CVD, which enabled a detailed investigation of their structural and electronic properties by various complementary experimental techniques. The experimental findings were supported by the results of DFT calculations. The formation of Janus SeMoS MLs was unambiguously confirmed by Raman spectroscopy and XPS, whereas the LEED measurements allowed us to precisely determine their lattice constant of 3.22 ± 0.01 Å. This value lies between the values of MoS_2_ and MoSe_2_ MLs and coincides with the theoretically predicted value. The observed moiré pattern of the Janus SeMoS ML on Au(111) with a moiré lattice constant of ∼2.9 nm is in line with the locally different charge transfer. The spin–orbit splitting of the valence band measured at the *K* point by ARUPS is 170 ± 15 meV and is in good agreement with the DFT predictions as well. To summarize, our experimental and theoretical results demonstrate that the Janus SeMoS ML is a direct band gap semiconductor, exhibiting structural and electronic properties that are intermediate between those of MoS_2_ and MoSe_2_ MLs. This study represents a significant step toward advancing the understanding of the structural and electronic characteristics of these novel 2D quantum materials.

## Supporting Information Available

Details of the growth and methods used for characterization, XPS fit details, DFT calculations, STM data, ARUPS data and analyses, and LEED data (PDF)
