# Supplementary material for: Atomic Structure and Electronic Properties of Janus SeMoS Monolayers on Au(111)
Source: Nano Lett. 2025 Feb 18;25(8):3330–6. doi: 10.1021/acs.nanolett.4c06543 (PMC11869296; doi:10.1021/acs.nanolett.4c06543)
Supplement: Supplementary file 1 — nl4c06543_si_001.pdf [file nl4c06543_si_001.pdf]

Supporting Information for

Atomic structure and electronic properties of Janus SeMoS monolayers on Au(111)

Julian Picker[1], Mahdi Ghorbani-Asl[2], Maximilian Schaal[3], Silvan Kretschmer[2], Felix Otto[3], Marco Gruenewald[3], Christof Neumann[1], Torsten Fritz[3,4], Arkady V. Krasheninnikov[2] and Andrey Turchanin[1,4*]

[1]*Institute of Physical Chemistry, Friedrich Schiller University Jena, 07743 Jena, Germany*

[2]*Institute of Ion Beam Physics and Materials Research, Helmholtz-Zentrum Dresden-Rossendorf, 01328 Dresden, Germany*

[3]*Institute of Solid State Physics, Friedrich Schiller University Jena, 07743 Jena, Germany*

[4]*Abbe Center of Photonics, Friedrich Schiller University Jena, 07745 Jena, Germany*

*Corresponding author: andrey.turchanin@uni-jena.de

Experimental

Sample preparation

Before the synthesis of the Janus SeMoS, we cleaned the Au(111) single crystal (MaTecK, 99.999%) by several steps of Ar^+ sputtering (1.0 keV) for 20 min and subsequent annealing at 580 °C for 30 min. These steps were repeated until no contaminations were detected by XPS and the (22 × √3) reconstruction of the Au(111) surface was visible by LEED. To create a small roughness on the substrate, we gently sputtered the Au(111) surface with Ar^+ ions (0.2 keV) for 5 min before the Janus synthesis. For the synthesis of the Janus SeMoS, a growth recipe similar to Ref.[1] was applied. Initially, $MoSe_2$ monolayers were grown by chemical vapor deposition in a two-zone furnace at ambient pressure. In the first zone, the Se pellets (Sigma Aldrich, 99.98%) were placed in a Knudsen cell and heated to 400 °C.[2] In the second zone, the MoO_3 powder (Sigma Aldrich, 99.97%) and the Au(111) single crystal were placed. The temperature of this zone was set to 750 °C for 15 min. As the carrier gas, a mixture of Ar (100 sccm) and H_2 (8 sccm) was employed. After the furnace cooled down, the Se Knudsen cell was exchanged with a Knudsen cell filled with S powder (Sigma Aldrich, 99.97%). Next, this zone was heated to 200 °C. The replacement of Se by S is more favorable for a Au(111) surface that has been slightly sputtered prior to the growth compared to a perfectly smooth Au(111) surface (see Figure S9). To achieve the conversion from $MoSe_2$ to Janus SeMoS, the temperature of the Au(111) substrate was kept at 700 °C for 15 min; 100 sccm of Ar was used as the carrier gas. Prior to characterization of the samples by ultrahigh vacuum methods, they were annealed *in situ* at 150 °C for 30 min. Thereby, atmospheric contaminations were removed from the sample.

Raman spectroscopy

Raman spectroscopy was measured at ambient conditions using a Bruker Senterra spectrometer operated in backscattering mode. A frequency-doubled Nd:YAG laser with a wavelength of 532 nm was used as excitation. Measurements were obtained with a 50x objective and a thermoelectrically cooled CCD detector. The spectral resolution of the system was 2-3 cm^{-1}.

Scanning tunneling microscopy

The following methods were carried out in UHV at a base pressure $< 3 \cdot 10^{-10}$ mbar. For scanning tunneling microscopy (STM) we used two different setups. A low-temperature STM (SPECS JT-STM/AFM) at 4.2 K with a Pt/Ir tip and a variable temperature STM (Scienta Omicron VT SPM) at room temperature (RT) with a W tip were used. All images were edited with Gwyddion (Version 2.56).[3] These include plane subtraction and fast Fourier transform (FFT).

Distortion-corrected low-energy electron diffraction

Low-energy electron diffraction (LEED) images were taken at RT by a single microchannel plate LEED system (SMCP, Scienta Omicron). These images were corrected from geometrical distortions and energy error using the LEEDCal software.[4, 5] Subsequently, the quantitative analysis of these images was performed with the LEEDLab software by fitting the visible LEED spots to a reciprocal structure.[6] The accuracy of this procedure is typically 1 %.[6] The quality of

the distortion correction and calibration of the LEED was checked by using the diffraction spots of the Au(111) substrate which are also visible in the LEED pattern. The Au(111) surface exhibit a lattice constant of 2.884 Å.[7]

X-ray photoelectron spectroscopy

X-ray photoelectron spectroscopy (XPS) was conducted using a monochromatic Al K_α X-ray source (1486.7 eV) and an electron analyzer with a spectral energy resolution of 0.6 eV (Argus CU). For analyzing the XP spectra, the software CasaXPS was employed. All spectra were calibrated to the Au $4f_{7/2}$ peak at a binding energy (BE) of 84.0 eV. Peaks were fitted by Voigt functions (Gaussian:Lorentzian = 70:30) after subtraction of a linear (S 2p, Se 3d) or Shirley (Mo 3d) background. For quantification of elemental ratios, Scofield's relative sensitivity factors of 1.11, 1.36 and 5.62 were used for the S $2p_{3/2}$, Se $3d_{5/2}$ and Mo $3d_{5/2}$ orbitals, respectively.[8] All XPS measurements were conducted at room temperature.

Angle-resolved ultraviolet photoelectron spectroscopy

Angle-resolved ultraviolet photoelectron spectroscopy (ARUPS) measurements were performed using a monochromatized, p-polarized He Iα (21.22 eV) excitation source (SPECS ultraviolet light source, UVLS) with an electron analyzer (SPECS PHOIBOS 150) providing an energy resolution of 60 meV. The band structure was recorded by tilting the sample to vary the photoemission angle. The binding energy scale of all UP spectra was calibrated to the Fermi edge of Au(111). For detailed band structure measurements, the 2D mode of the detector was utilized. All ARUPS measurements were conducted at room temperature.

Density functional theory calculations

Density functional theory (DFT) calculations were carried out using the Vienna Ab initio Simulation Package (VASP), based on the plane-wave projector augmented-wave (PAW) method.[9, 10] The exchange-correlation functional was employed in the generalized gradient approximation of Perdew-Burke-Ernzerhof.[11] Van der Waals (vdW) interactions were taken into account using the Grimme (DFT-D3) method,[12] which provided a compromise between accuracy and computational efficiency as compared to more sophisticated methods.[13-17] Test calculations using DFT-D2 scheme gave essentially the same results.[16, 17] For the plane-wave expansion of the primitive cell and supercell calculations, energy cut-offs of 400 eV were chosen, as the calculations with higher cutoff energies gave essentially the same results. The effect of spin-orbit coupling is included in the electronic structure calculations. The SeMoS/Au(111) heterostructure was constructed by using an interface consisting of (10 × 10) unit cells of Au (111) and (9 × 9) unit cells of a SeMoS monolayer corresponding to a lattice mismatch of only 0.13%. The supercell has a lattice constant of $a = b = 28.99$ Å including 643 atoms. The Brillouin zone of the supercells was sampled using Gamma-point approximation, and a (12 × 12) mesh was used for the primitive cell. The charge densities were visualized using the VESTA package.[18] Constant current STM images were simulated using the Tersoff–Hamann approximation.[19]

Table S1: Peak position, full width-at-half-maximum (FWHM) and the composition from the Mo 3d, S 2p and Se 3d spectra presented in Fig. 1c.

**Peak Assignment**	**Binding energy, eV**	**FWHM, eV**	**Composition, at%**
Janus SeMoS on Au(111), 0°			
Mo $3d_{5/2}$ (SeMoS)	228.9(1)	0.5(1)	18(2)
Mo $3d_{3/2}$ (SeMoS)	232.0(1)	0.7(1)	
Mo $3d_{5/2}$ ($MoSe_2$)	229.3(1)	0.6(1)	5(2)
Mo $3d_{3/2}$ ($MoSe_2$)	232.5(1)	0.6(1)	
Mo $3d_{5/2}$ (MoO_3)	231.0(2)	3.0(3)	7(2)
Mo $3d_{3/2}$ (MoO_3)	234.2(2)	3.5(3)	
Se 3s	229.3(2)	1.8(3)	-
S 2s	226.9(1)	1.5(3)	-
S $2p_{3/2}$ (SeMoS)	162.2(1)	0.8(1)	21(2)
S $2p_{1/2}$ (SeMoS)	163.4(1)	0.8(1)	
S $2p_{3/2}$ (S)	163.4(2)	1.2(2)	11(2)
S $2p_{1/2}$ (S)	164.5(2)	1.2(2)	
Se $3p_{3/2}$	160.9(1)	1.8(1)	-
Se $3p_{1/2}$	166.6(1)	2.0(1)	
Se $3d_{5/2}$ (SeMoS, $MoSe_2$)	54.5(1)	0.6(1)	38(2)
Se $3d_{3/2}$ (SeMoS, $MoSe_2$)	55.4(1)	0.6(1)	
Au $5p_{3/2}$	57.0(3)	5.0(5)	-
Mo 4s	62.3(3)	4.0(5)	-
Janus SeMoS on Au(111), 70°			
Mo $3d_{5/2}$ (SeMoS)	228.8(1)	0.5(1)	19(2)
Mo $3d_{3/2}$ (SeMoS)	232.0(1)	0.7(1)	
Mo $3d_{5/2}$ ($MoSe_2$)	229.3(1)	0.7(1)	5(2)
Mo $3d_{3/2}$ ($MoSe_2$)	232.4(1)	0.7(1)	
Mo $3d_{5/2}$ (MoO_3)	231.0(2)	3.0(3)	8(2)
Mo $3d_{3/2}$ (MoO_3)	234.2(2)	3.5(3)	
Se 3s	229.2(2)	1.8(3)	-
S 2s	226.8(1)	1.7(3)	-
S $2p_{3/2}$ (SeMoS)	162.3(1)	0.8(1)	12(2)
S $2p_{1/2}$ (SeMoS)	163.6(1)	0.8(1)	
S $2p_{3/2}$ (S)	163.4(2)	1.2(2)	16(2)
S $2p_{1/2}$ (S)	164.5(2)	1.2(2)	
Se $3p_{3/2}$	160.9(1)	2.0(1)	-
Se $3p_{1/2}$	166.5(1)	2.3(1)	
Se $3d_{5/2}$ (SeMoS, $MoSe_2$)	54.5(1)	0.6(1)	40(2)
Se $3d_{3/2}$ (SeMoS, $MoSe_2$)	55.4(1)	0.6(1)	
Au $5p_{3/2}$	57.0(3)	4.8(5)	-
Mo 4s	62.8(3)	4.0(5)	-

Table S2: Calculated lattice parameters (Å), bond lengths (Å) and bond angles (°) in MoS_2, SeMoS and $MoSe_2$ MLs.

Material	*a*	Mo-S	Mo-Se	S-Mo-S	S-Mo-Se	Se-Mo-Se
MoS_2	3.15	2.40	-	81.51	-	-
SeMoS	3.22	2.41	2.52	-	82.23	-
$MoSe_2$	3.28	-	2.53	-	-	82.98

Table S3: Atomic charges (Δq_{Mo}, Δq_{S}, Δq_{Se}; Δq = q (atom in solid) – q (isolated atom)) in MoS_2, SeMoS and $MoSe_2$ MLs.

Material	Δq_{Mo}	Δq_{S}	Δq_{Se}
MoS_2	1.04	-0.52	-
SeMoS	0.91	-0.56	-0.35
$MoSe_2$	0.73	-	-0.36

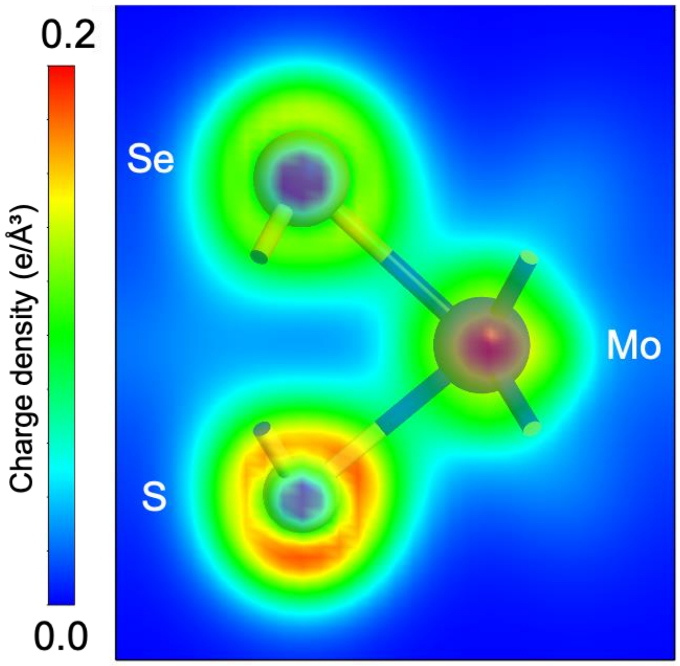

Figure S1: Calculated contour plots of the charge density at SeMoS ML.

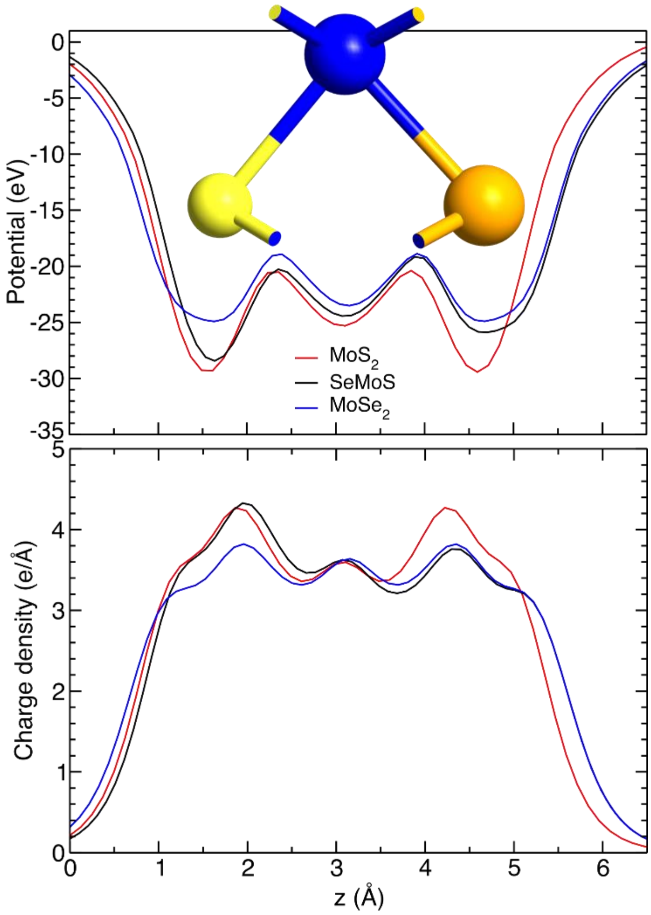

Figure S2: Averaged electrostatic profile and charge densities along the perpendicular (z) direction to the MoS_2, SeMoS and $MoSe_2$ MLs. The densities were averaged in the x-y plane and presented vs. z-coordinate.

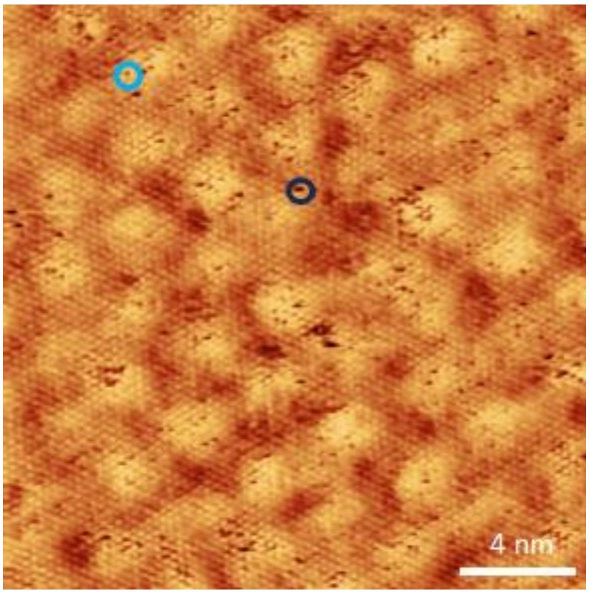

Figure S3: Atomically resolved STM image of a Janus SeMoS ML on a Au(111) surface (0.8 V, 1.0 nA, 293 K). Defects, probably chalcogen vacancies, are visible. The defect density is in the order of 10^{12} cm^{-2}. Different types of vacancies can be identified: Single vacancies (blue) or multi vacancies (black), where many atoms are missing.

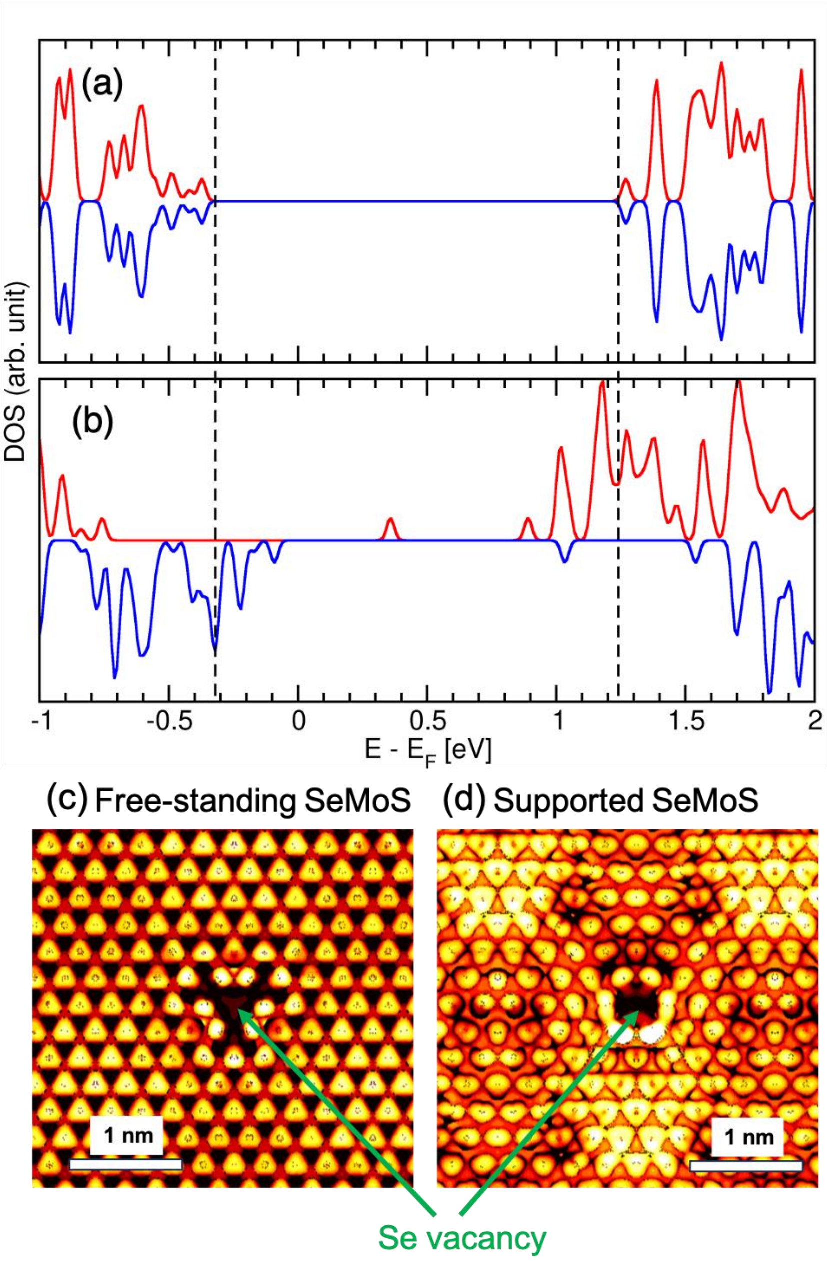

Figure S4: Calculated density of states of a free-standing (a) pristine and (b) defective SeMoS ML. Blue and red indicate spin up and spin down states. The highest occupied state in the defective material is set to zero. The band edges of the pristine SeMoS are indicated by the black dashed lines. (a, b) Simulated STM images of a Se vacancy for both (c) a free-standing and (d) a gold supported SeMoS ML. As evident from the DOS plot, pristine SeMoS is non-magnetic whereas defective SeMoS has magnetic moments localized at defects.

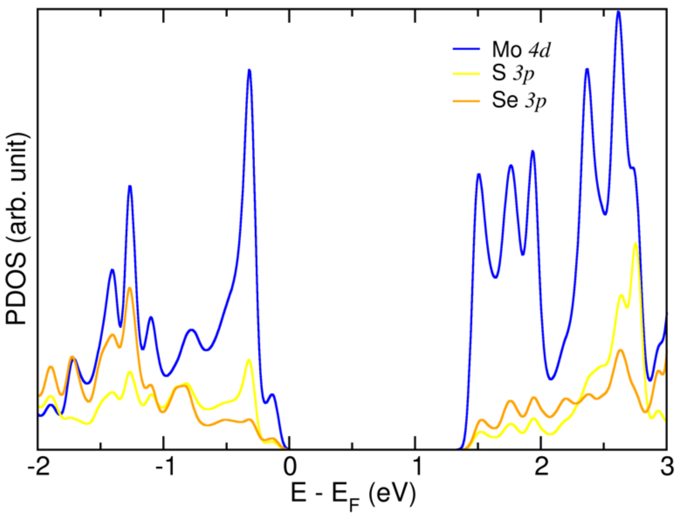

Figure S5: Projected density of states (PDOS) of a free-standing Janus SeMoS ML.

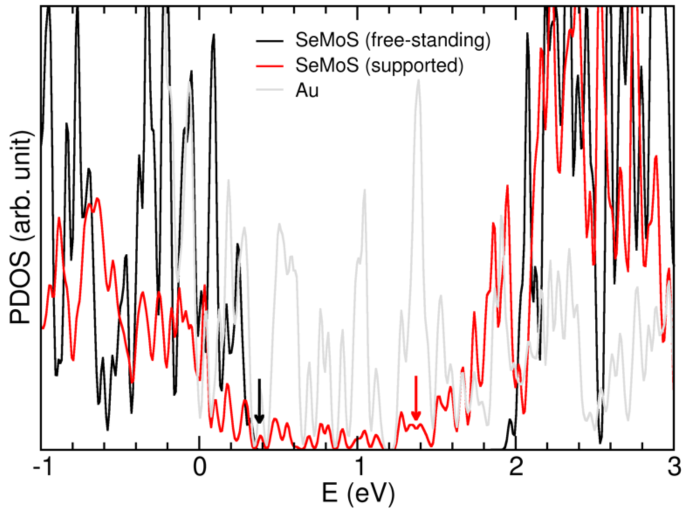

Figure S6: Projected density of states (PDOS) for a SeMoS ML supported on a Au(111) substrate. The density of states of a free-standing SeMoS ML is given for comparison. The arrows indicate the highest occupied state in free-standing and supported SeMoS.

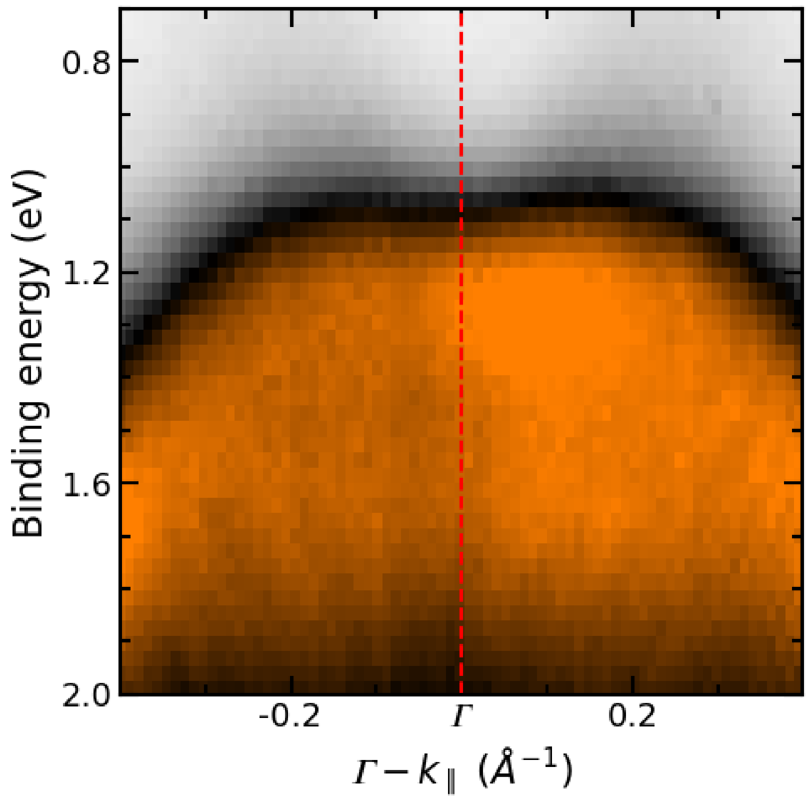

Figure S7: ARUPS data of Janus SeMoS monolayers on Au(111) around the *Γ* point.

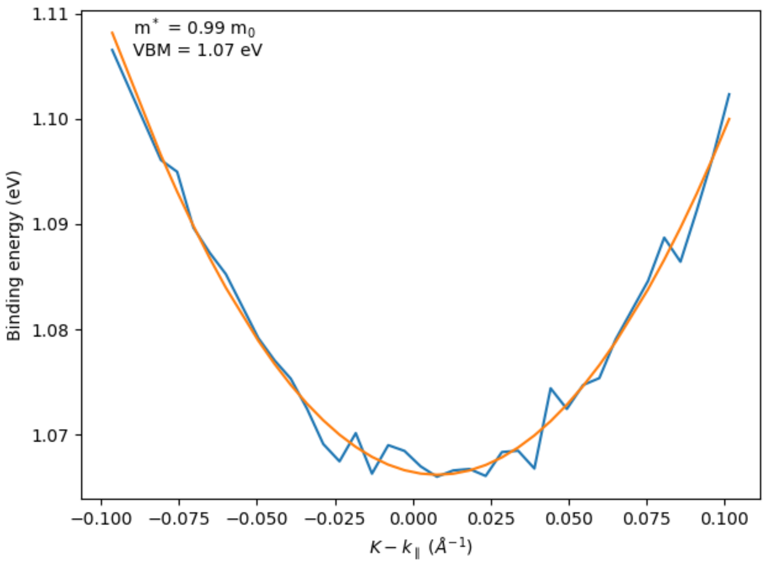

Figure S8: Fit of the valence band around the *K* point shown in Figure 4b. For each energy distribution curve (constant *k* value) the position of the valence band in binding energy is fitted by a gaussian function. The valence band maximum (VBM) and the effective mass (m*) are obtained from a parabola fit (orange).

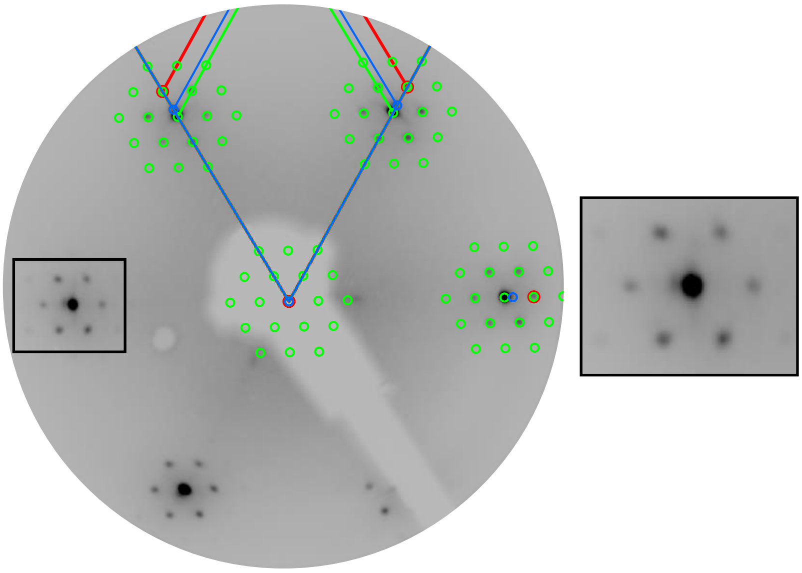

Figure S9: LEED pattern of MoS_2 (blue) and $MoSe_2$ (green) MLs on Au(111) (red). We identified two different LEED patterns. From quantitative LEED analysis, we found that these structures match the lattice constants of MoS_2 and $MoSe_2$. We did not find the Janus structure. This sample was grown with the same parameters as the sample discussed in the main paper (cf. Figure 2a). However, the difference in both samples lies in the preparation of the Au(111) substrate. The Au(111) crystal was annealed at around 600 °C for 30 min in UHV as the last preparation step and then introduced into the CVD chamber. For the sample which we discussed in the main paper, we moderately sputtered the Au(111) crystal with Ar^+ ions to introduce roughness into the Au(111) surface. This roughness is necessary for the conversion from $MoSe_2$ to a Janus structure.

References

1. Gan, Z.; Paradisanos, I.; Estrada‐Real, A.; Picker, J.; Najafidehaghani, E.; Davies, F.; Neumann, C.; Robert, C.; Wiecha, P.; Watanabe, K., Chemical Vapor Deposition of High‐Optical‐Quality Large‐Area Monolayer Janus Transition Metal Dichalcogenides. *Adv. Mater.* **2022,** *34* (38), 2205226.
2. George, A.; Neumann, C.; Kaiser, D.; Mupparapu, R.; Lehnert, T.; Hübner, U.; Tang, Z.; Winter, A.; Kaiser, U.; Staude, I., Controlled growth of transition metal dichalcogenide monolayers using Knudsen-type effusion cells for the precursors. *J. Phys. Mater.* **2019,** *2* (1), 016001.
3. Nečas, D.; Klapetek, P., Gwyddion: an open-source software for SPM data analysis. *Open Phys.* **2012,** *10* (1), 181-188.
4. Sojka, F.; Fritz, T., LEEDCal 2013, version 4.0, Fritz & Sojka GbR, Apolda Germany. **2018**.
5. Sojka, F.; Meissner, M.; Zwick, C.; Forker, R.; Fritz, T., Determination and correction of distortions and systematic errors in low-energy electron diffraction. *Rev. Sci. Instrum.* **2013,** *84* (1), 015111.
6. Sojka, F.; Fritz, T., LEEDLab 2018, version 1.0, Fritz & Sojka GbR, Apolda Germany. **2018**.
7. Dutta, B.; Dayal, B., Lattice Constants and Thermal Expansion of Gold up to 878° C by X‐Ray Method. *Phys. Status Solidi B* **1963,** *3* (3), 473-477.
8. Scofield, J. H., Hartree-Slater subshell photoionization cross-sections at 1254 and 1487 eV. *J. Electron. Spectrosc. Relat. Phenom.* **1976,** *8* (2), 129-137.
9. Kresse, G.; Furthmüller, J., Efficiency of ab-initio total energy calculations for metals and semiconductors using a plane-wave basis set. *Comput. Mater. Sci.* **1996,** *6* (1), 15-50.
10. Kresse, G.; Furthmüller, J., Efficient iterative schemes for ab initio total-energy calculations using a plane-wave basis set. *Phys. Rev. B* **1996,** *54* (16), 11169.
11. Perdew, J. P.; Burke, K.; Ernzerhof, M., Generalized gradient approximation made simple. *Phys. Rev. Lett.* **1996,** *77* (18), 3865.
12. Grimme, S.; Mück-Lichtenfeld, C.; Antony, J., Noncovalent Interactions between Graphene Sheets and in Multishell (Hyper)Fullerenes. *J. Phys. Chem. C* **2007,** *111* (30), 11199-11207.
13. Steinmann, S. N.; Corminboeuf, C., Comprehensive benchmarking of a density-dependent dispersion correction. *J. Chem. Theory Comput.* **2011,** *7* (11), 3567-3577.
14. Caldeweyher, E.; Ehlert, S.; Hansen, A.; Neugebauer, H.; Spicher, S.; Bannwarth, C.; Grimme, S., A generally applicable atomic-charge dependent London dispersion correction. *J. Chem. Phys.* **2019,** *150* (15).
15. Ambrosetti, A.; Reilly, A. M.; DiStasio, R. A.; Tkatchenko, A., Long-range correlation energy calculated from coupled atomic response functions. *J. Chem. Phys.* **2014,** *140* (18).
16. Halbertal, D.; Finney, N. R.; Sunku, S. S.; Kerelsky, A.; Rubio-Verdú, C.; Shabani, S.; Xian, L.; Carr, S.; Chen, S.; Zhang, C., Moiré metrology of energy landscapes in van der Waals heterostructures. *Nat. Commun.* **2021,** *12* (1), 242.
17. Rawat, A.; Mohanta, M. K.; Jena, N.; Dimple; Ahammed, R.; De Sarkar, A., Nanoscale interfaces of Janus monolayers of transition metal dichalcogenides for 2D photovoltaic and piezoelectric applications. *J. Phys. Chem. C* **2020,** *124* (19), 10385-10397.
18. Momma, K.; Izumi, F., VESTA: a three-dimensional visualization system for electronic and structural analysis. *J. Appl. Crystallogr.* **2008,** *41* (3), 653-658.
19. Tersoff, J.; Hamann, D. R., Theory of the scanning tunneling microscope. *Phys. Rev. B* **1985,** *31* (2), 805.
